# Influence of Education

**Published:** 1869-11

**Authors:** 


### Selections.

---

INFLUENCE OF EDUCATION.—Dr. Brakenridge, in an article in the *Times and Gazette*, supports the following thesis:

The wider the range of the education, the more vigorous will be the health, and the greater the range of circumstances over which it may be preserved.

Were the constitution limited to one constant unvarying set of influences, health would be incompatible with the slightest deviation from this. The provisions of nature secure a considerable variety in this respect. In the rotation of the seasons we have a constant succession of changes which check the tendency of the body to fall into a too narrow series of habitual activities. The width of education thus afforded renders it possible to move with comfort and safety to considerably distant localities at seasons when their climate approaches in character to one or other of the seasons in the native climate. Thus we may indulge in a change to colder places during their summer if it is not colder than our winter, and to warmer places during their winter if it is not warmer than our summer. It is evident that the extent of this capacity for change must be regulated by the range of conditions to which the body has been accustomed, being greater or less according to the variability or equability of the developing climate. The development brought about by any one locality must, however, be, at the best, very limited compared with man's capacity. Doubtless the craving for change which man feels with regard to many things is not without its physiological importance.

Such deep-seated yearnings as the love of travel, and the great inventions to which, for its satisfaction, it stimulates him, hint that this local development, even where the balance of functional power is best preserved, by no means represents the highest state which he is capable of attaining. He possesses, in a rudimentary condition it may be, but none the less truly, powers which, were they all fully educated, might render him cosmopolitan. Greatly increased rapidity of locomotion is already doing much to extend the range of his physiological education, and, in addition, the blending of different races which is thus favored, and the consequent intermarriages of those possessing constitutions widely different, will hasten the higher development of the whole race, For when we have the organs which in the one parent are highly trained, uneducated in the other, and *vice versa*, it is reasonable to suppose that the positive and not the negative, properties of each parent will have the greater tendency to be transmitted to the offspring; hence it will possess somewhat of the special powers and capacities of both. Thus the offspring of aboriginal and European parents in Hindostan is said to "inherit from the native parent a certain adaptation to the climate, and from the European a higher development of brain." (Combe's Constitution of Man," pp. 194). Many examples might be adduced to show that mixed races of men surpass in vigor and in the tendency to multiply the parent races from which they have sprung. It must to a considerable extent be regarded as the explanation of the high vigor of the Anglo-Saxon race that in it there are blendid together so many constitutions, originally very differently educated, and consequently possessing widely different powers.

